# Rapid public health genomics capacity building during the COVID-19 Delta crisis driven by a transnational private, public, and social alliance

**DOI:** 10.3389/fpubh.2025.1602152

**Published:** 2025-07-17

**Authors:** Mehul Champaklal Mehta, Gayatri Nair Lobo, Gretchen Weightman, Vidushi Chitalia, Jayanthi Shastri

**Affiliations:** ^1^Albright Stonebridge Group, Washington, DC, United States; ^2^Department of Ophthalmology, Harvard Medical School, Boston, MA, United States; ^3^School of Public Health, Yale University, New Haven, CT, United States; ^4^ATE-Chandra Foundation, Mumbai, India; ^5^Educate Girls, Mumbai, India; ^6^Illumina Corporation, San Diego, CA, United States; ^7^Oxford Nanopore Technologies, Oxford, United Kingdom; ^8^GeSCOPE, Kasturba Hospital, Municipal Corporation of Greater Mumbai, Mumbai, India; ^9^Indian Institute of Technology Bombay, Mumbai, India; ^10^Department of Microbiology and Immunology, Topiwala National Medical College, Municipal Corporation of Greater Mumbai, Mumbai, India; ^11^Maharashtra University of Health Sciences, Nashik, India

**Keywords:** public health genomics, COVID-19, public private social partnerships, capacity building, survellience system, lessons learnt

## Abstract

When the COVID-19’s Delta variant spread globally during late 2020, Public Health Genomics (PHG) capabilities were missing in several cities, significantly impairing the ability of policymakers and public health systems to rapidly identify COVID-19 variants and effectively respond, with tragic human and economic consequences. In this community case study, we retrospectively studied the dire situation during the Delta wave 5 years ago when we rapidly constituted a transnational public, private, and social, pro-bono alliance to establish PHG capabilities in Mumbai, a mega metropolis that lacked this capacity. The Albright Stonebridge Group—a renowned global commercial diplomacy entity, Illumina—a genomic sequencing equipment leader, a leading Mumbai non-governmental organization—the ATE Chandra Foundation, and the Municipal Corporation of Greater Mumbai, came together in an alliance to rapidly establish PHG capabilities in Mumbai. This effort was coordinated across multiple countries, with distributed decision-making and defined responsibilities. An optimal site for a PHG center in Mumbai was identified, and the operational requirements, governance structures, resource and sustainability requirements were defined. In 3 months, the Genome Sequencing Centre for Outbreak Preparedness & Epidemiology (GeSCOPE) was successfully established in Mumbai’s public infectious disease hospital, Kasturba. The percentage of samples sequenced increased from less than 1% to over 30%, including all known RT-PCR positive cases, the sequencing time dropped to under 72 h, and public health policy and responses were enhanced. Currently, GeSCOPE has established itself as the epidemiological hub for Mumbai and western India at large, identifying infectious disease threats such as Dengue, Chikungunya, and drug resistance genes in *Mycobacterium tuberculosis* strains. This has transformed Mumbai’s ability to expeditiously identify and contain infectious outbreaks, thus better protecting its citizens. Our experience has generalizable lessons that we hope will inform other communities seeking to establish and strengthen their PHG capabilities.

## Introduction

The COVID-19’s Delta variant (B.1.617.2) that ravaged India and the world originated in the state of Maharashtra in October 2020, close to the city of Mumbai, India’s economic and international hub (1). This variant was identified late, leading to a significant spike in infections and mortality (2). Healthcare systems and professionals were stretched to their limit, and the national and state governments were in overdrive, trying to cope with this unprecedented national crisis. It is estimated that during the spring of 2021, the average death rate in India doubled from 3 to 6% in less than 3 months (3).

It became critical to understand why the Delta variant was not quickly identified and contained in Mumbai. Urgent questions around measures that could be rapidly adopted to prevent another highly pathogenic variant from spreading had to be addressed immediately. Many other cities and countries faced a similar situation.

Rapid identification of a pathogen through a pathogen genomics surveillance system is vital to contain the spread of any infectious outbreak. Identification of a pathogen and its variants is the critical first step (4). The ability to locally and expeditiously carry out advanced whole-genome sequencing of a suspected pathogen is now recognized as a key component of any pandemic prevention and public health response system. Without this capacity in place, health professionals, governments, and public health systems cannot make informed decisions (5). We, therefore, set out to rapidly establish pathogenic whole-genome sequencing capacity in Mumbai’s public health system to better protect the city’s citizens and the national and global communities.

## Methods

### Understanding the context

As the COVID-19’s Delta variant (B.1.617.2) spread across India with devastating consequences, the health systems group at the Albright Stonebridge Group (ASG) decided to establish Public Health Genomics (PHG) capabilities in Mumbai, as a pro bono effort, as soon as possible.

It was important to first understand the current context of PHG sequencing capabilities for COVID-19 in India. Before the pandemic, genomic sequencing in India was primarily a research endeavor, limited to select national institutes in a few cities. These centers were neither specifically tailored to conduct public health genomic surveillance of infective pathogens, nor were they fully integrated with regional public health systems to help guide public health responses. This situation was not unique to Mumbai. Many Indian cities faced a similar situation, as do other cities globally.

During the COVID-19 pandemic in late 2020, the Government of India pivoted these national research institutes to focus on whole-genome sequencing of positive COVID-19 samples, offering some genomic surveillance support to regional public health systems. While this was an essential pivot, it posed challenges in supporting the timely and effective surveillance needs of many cities in India. Mumbai, a mega-metropolis of 21 million inhabitants, is no exception. The closest center designated to support Mumbai’s COVID-19 genomic surveillance needs was the National Institute of Virology (NIV), primarily a research institute, located 150 kilometers east of Mumbai. Only a small fraction (<1%) of reverse transcription-polymerase chain reaction (RT-PCR) positive COVID-19 samples from Mumbai were sent to NIV for sequencing. This problem was compounded by the logistical challenges of transporting samples from Mumbai to Pune over such a distance. The time lag, often in days, to acquire results, identify super-spreaders, and understand which COVID-19 variants were active, significantly undermined public health responses, effective containment efforts, and public health policy decisions.

It is in this context that the COVID-19’s Delta variant (B1.617.2) emerged close to Mumbai, in late 2020. However, its identification and containment were delayed, leading to a national and global spread, with unfortunate human and economic consequences.

### Establishing the alliance

It was apparent that rapidly establishing public health COVID-19 genomic sequencing capacity in Mumbai needed to be a priority. However, to do so successfully, it was also essential to establish a global alliance of entities that were committed to the same purpose and who could contribute to the critical elements needed, such as providing advanced genomic sequencers for use in public health settings, funding, and human resources support, coordination on the ground and obtaining the support of the Municipal Corporation of Greater Mumbai (MCGM).

Illumina, a California-based global manufacturing leader in advanced genomic sequencers, was a natural choice. Illumina’s sequencers were already being used by India’s national research institutes. Illumina had also worked with the Government of India to help establish India’s SARS-CoV-2 Genomics Consortium (INSACOG), a multi-laboratory, multi-agency, national network to monitor genomic variations in SARS-CoV-2. ASG’s team believed that if Illumina’s leadership agreed to donate their next-generation genomic sequencers to the city of Mumbai, as part of their Corporate Social Responsibility (CSR) efforts, then along with the help of the right social sector non-governmental organizations (NGOs) in Mumbai, critical support of the city’s municipal leaders could be obtained for this vital endeavor.

An urgent advocacy effort at the level of Illumina’s leadership was launched by ASG’s team. At this time, the Delta variant was rapidly spreading in India with devastating outcomes. Hence, despite unprecedented global demand for their sequencers, Illumina’s leadership and its Board decided to participate in the alliance and, as a special CSR effort, donate two advanced Illumina NextSeq 2000 sequencer systems, with a capacity of sequencing 376 COVID-19 positive samples in one run. Illumina also agreed to support the cost of the initial supplies for sequencing and train the key technical personnel on their sequencers, to ensure operational readiness.

ASG then turned to identify the right social NGO partner in Mumbai. The A. T. E. Chandra Foundation (ATECF) in Mumbai was chosen as the ideal entity to manage the project on the ground and engage with the MCGM. At the outset, ATECF understood Mumbai’s public health system and was respected by both the MCGM and Mumbai’s public health officials. They had already responded to the pandemic, identified public health gaps, and provided immediate assistance to the MCGM. ATECF had also initiated India’s first and largest Metropolitan COVID-19 serosurveys, which provided invaluable insights to policymakers. Finally, ATECF also expressed its willingness to reach out to other NGOs and global multilateral agencies and raise the needed funding to support operations in the first few years. ATECF’s Board committed to the alliance and took the lead in reaching out to public health leaders in the city of Mumbai and the MCGM. They quickly obtained strong support from key stakeholders bringing them into the alliance.

### Identifying the ideal site

While establishing an alliance was a promising start, it became apparent that in the absence of ‘system readiness’ to incorporate genomic sequencing into Mumbai’s public health system effectively and sustainably, the entire endeavor would fail. To address this key issue, ASG’s team took a two-pronged approach: With ATECF’s help, they first conducted an objective assessment of the most ‘system-ready’ sites in Mumbai. After exploring several options, it became apparent that Mumbai’s public Kasturba Hospital (KH) was the optimal site to house the sequencers and establish Mumbai’s PHG center. The reasons were compelling. KH was the only dedicated public infectious diseases hospital in Mumbai. It was also integrated with the city’s public health system, conducted infectious outbreak surveillance, had a well-established Molecular Diagnostic & Reference Laboratory (MDRL), and was a designated Viral Research & Diagnostic Laboratory (VRDL) for the Indian Council of Medical Research’s (ICMR). In early 2020, KH was designated by ICMR as one of the 13 laboratories authorized nationally to conduct COVID-19 tests. In early 2020, KH’s VRDL, was already testing over 500 samples per day from Mumbai’s public and private sector hospitals and labs for RT-PCR testing. Finally, KH was part of the city’s municipal teaching hospital system, with a highly experienced faculty of microbiologists, virologists, infectious diseases specialists, and a trained staff of laboratory technicians.

Secondly, four transnational teams were rapidly formed: ASG in Boston and Washington, DC, Illumina in Singapore, San Francisco, Auckland, and Mumbai, and ATECF and MCGM in Mumbai. One representative from each team took the lead. Roles and responsibilities were identified based on each entity’s strengths and core competence, as follows:

- ASG would coordinate across all the alliance members, work with Illumina’s leadership and teams to help land their NextSeq 2000 sequencer systems in Mumbai, and work with ATECF and MCGM to house the sequencers in KH.
- Illumina would donate and airlift the sequencers to Mumbai, train the local personnel, and provide the initial operating supplies.
- ATECF, working with Illumina and ASG, would build a detailed business, funding, and sustainability plan, define the operating resource needs, commit to contributing 25% of the operating capital, and raise the balance to cover the first 5 years of operating costs. They would also take the lead in working with KH and MCGM, project managing the effort on the ground.
- MCGM would be responsible for and run the sequencers at KH. They would provide the site for the PHG center, adjacent to their molecular diagnostic laboratory at KH, build the physical infrastructure needed to house the sequencers, provide the required personnel, and select the leadership to run the PHG center. They would also integrate the center into Mumbai’s public health system and consider supporting the operating costs after 5 years, through budgetary allocations from the city’s health budget.

Once the roles were defined and key responsibilities were assigned, alliance members worked at an intense pace, across multiple countries and time zones, managing complex and interrelated workstreams. ATECF managed to quickly bring in additional funders—Give India, Godrej Seeds and Genomics, Ltd., and the Bill & Melinda Gates Foundation.

### Overcoming hurdles

On May 14, 2021, Illumina informed the group that they had received clearance from their Board to donate two Illumina NextSeq 2000 sequencer systems, with COVID-19-specific modules, and other supporting equipment, under their CSR program. However, immediate regulatory challenges surfaced. Even though the sequencers were being provided as a donation, they were not exempt from Indian import taxes. There were also regulatory restrictions related to accepting foreign equipment donations. Working through these challenges could have caused major delays, directly impacting Mumbai’s ability to respond to the ongoing crisis. In addition, the enormous global demand for sequencers also meant that any delay would significantly impact Illumina’s ability to fulfill its commitment to donate the sequencers.

Quickly overcoming these hurdles became a critical priority. ATECF’s and MCGM’s team leaders, with support from legal experts, researched, identified, and demonstrated to the Indian regulatory authorities legal clauses exempting MCGM from foreign equipment donation restrictions, under certain circumstances. However, quickly solving the import tax burden or rapidly soliciting additional funds to cover this tax liability proved to be extremely challenging. Fortunately, at this stage, Illumina’s leadership stepped in and decided to cover the entire Indian import tax liability as an additional CSR effort.

## Results and outcomes

### Impact of genome sequencing Centre for Outbreak Preparedness and epidemiology (GeSCOPE)

On 29 July 2021, 3 months after ASG’s team initiated action, Mumbai had its first PHG center at KH. This center was quickly integrated into the city’s public health and infectious outbreak prevention system, leading to the establishment of the Genome Sequencing Centre for Outbreak Preparedness & Epidemiology, GeSCOPE. In October 2021, GeSCOPE also became part of India’s SARS-CoV-2 Genomics Consortium, INSACOG, the national genomics network on infectious diseases monitoring and surveillance. The establishment of GeSCOPE rapidly changed Mumbai’s infectious outbreak management and pandemic preparedness capabilities.

**Figure 1:**
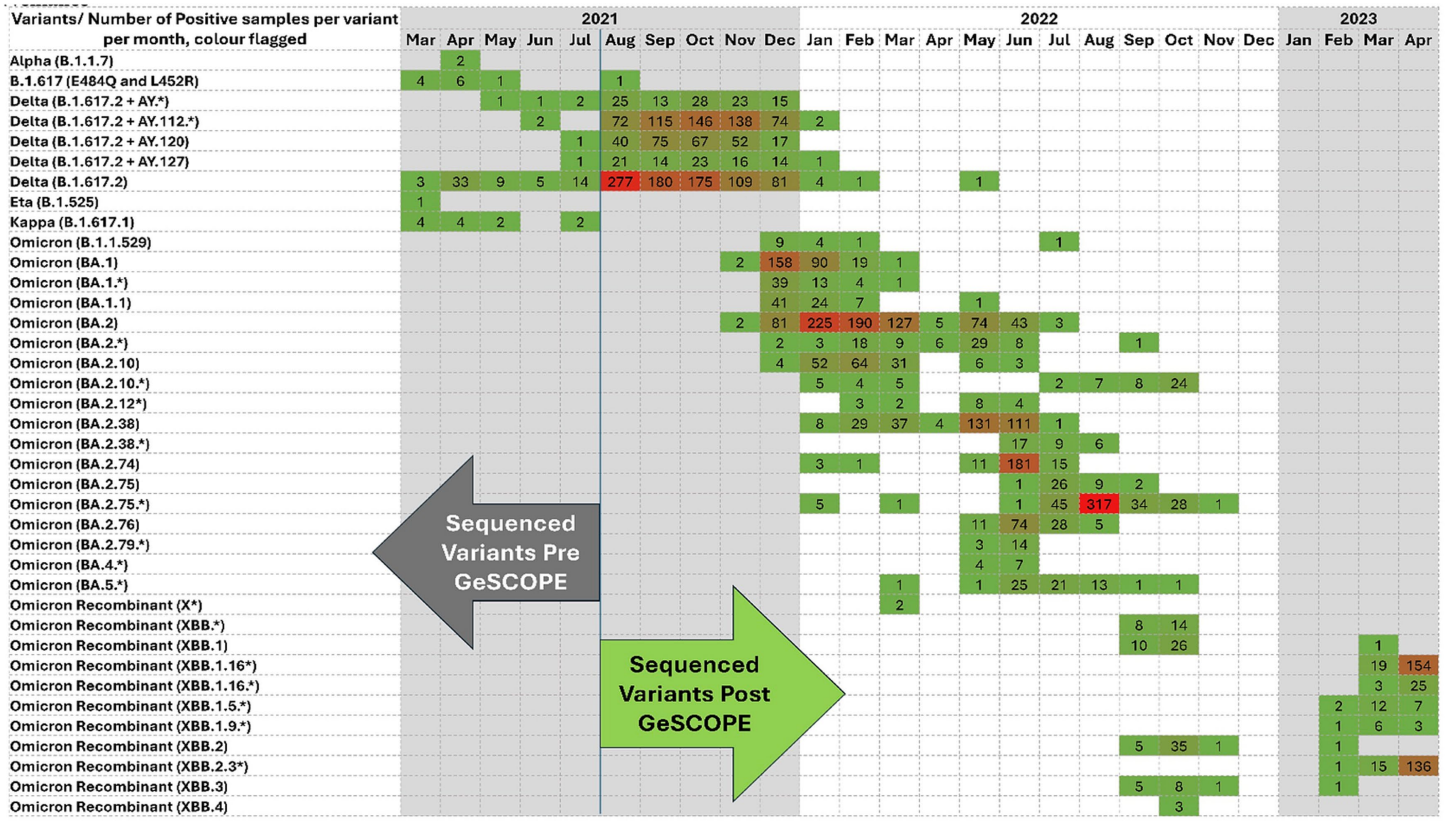
COVID variant sequencing heatmap demonstrating the evolution of SARS-CoV-2 lineages from March 2021 to April 2022 from March 2021 to April 2023. Each cell represents a positive sample for a specific variant/month. The color intensity indicates the frequency. Darker shades of Red reflect a higher case count; green indicates a lower case count. The figure shows the transition from early variants, the Delta and Delta derivatives, to the Omicron sub-lineages and recombinants, and the dynamic nature of viral evolution. The arrows denote the sequencing data before and after GeSCOPE’s establishment. (*) indicates the sub-variant of the particular variant.

**Figure 2:**
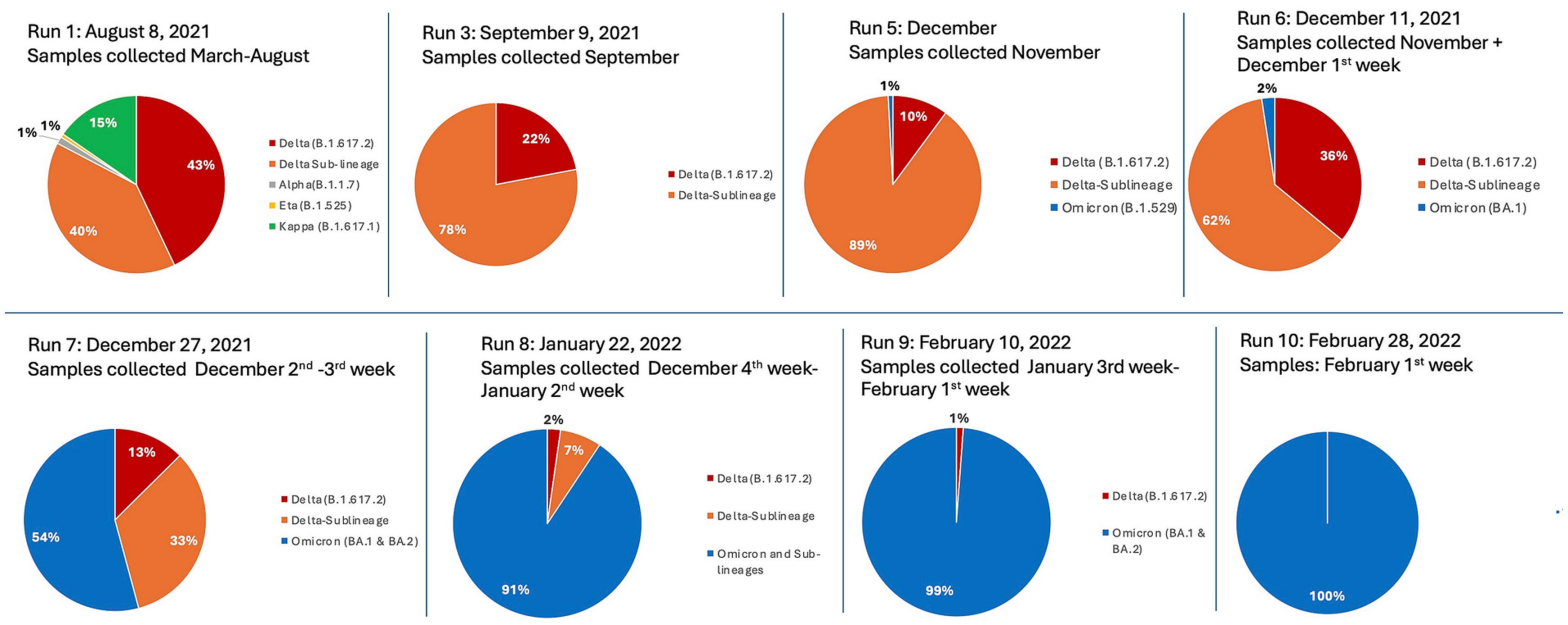
Defined time-series sequencing runs, representing GeSCOPE’s identification and tracking of the relative distribution of major SARS-CoV-2 variants from March 2021 to February 2022. The chronology shows an evolution from dominantly Delta and its sub-lineages (denoted as orange and red) in 2021, to dominantly Omicron and its sub-lineages (denoted as blue), from December 2021 onwards. Minor early variants such as Alpha, Eta, and Kappa are also represented. Note, runs 2 and 4 are not included, as their results were nearly identical to run 3.

GeSCOPE’s impact was significant, compared to the situation before its establishment (Figure 1). Today, sequencing is done free of charge at GeSCOPE. Metadata, including variant information, is compiled in MCGM’s public health epidemiology cell. As of late 2024, GeSCOPE had sequenced the majority of the COVID-19 RT-PCR-positive samples, plus sewage sampling, allowing for the establishment of an effective wastewater surveillance system in Mumbai. Armed with sequencing capabilities, Mumbai managed Omicron and its lineages effectively, compared to when PHG capabilities were absent. Variant-specific information has been very valuable in understanding the clinical and epidemiological trajectory of COVID-19 (Figure 2).

GeSCOPE has been able to achieve the following unique outcomes:

- Ascertaining and tracking the current status of new variants of SARS-CoV-2 in Mumbai, including the Delta derivatives, Omicron, and its lineages, and mapping them (Figure 2). These data from GeSCOPE have been of significant value locally, nationally, and even globally to track the evolution of SARS-CoV-2.
- Establishing a sentinel surveillance system for the early detection of genomic variants and marrying them to public health actions.
- Determining the genomic variants in unusual events/trends (super-spreader events, high mortality/morbidity trend analysis, etc.).
- Understanding the impact of emerging variants on clinical severity, transmissibility, efficacy of therapies, mortality, and morbidity.
- Sequencing of sewage samples to understand the presence of impending variants that could potentially cause outbreaks soon.
- Informing policy with empirical data and enabling policymakers at the MCGM to make appropriate decisions and adequately plan interventions.
- Expanding pathogen sequencing beyond COVID-19 to Dengue, Chikungunya, the Human Papillomaviruses, and more recently the detection of antimicrobial resistance genes in multidrug-resistant organisms, including in *Mycobacterium tuberculosis* strains.

### Current operating metrics

The following are the current operating metrics of GeSCOPE:

- *Sampling Rate*: Following its establishment, GeSCOPE received 100% of the known RT-PCR positive samples with cycle threshold values <30, from the public and private hospitals, community clinics, and all the laboratories in Mumbai, for sequencing and variant identification. The sampling rate jumped from under 1% before GeSCOPE’s establishment to over 50% in early 2024.
- *Costs:* Initial consumables and kits required for sequencing were provided by Illumina. Subsequent consumables and kits for all human and sewage sample sequencing were provided by ATECF. Hence, there has been no financial burden on KH and MCGM for the last 5 years. Sequencing was, and still is, provided free of cost. Today, GeSCOPE is developing a multipronged funding support model to diversify its funding sources.
- *Volume and Turnaround Time:* Since its establishment, GeSCOPE has sequenced 7,000 COVID-19 RT-PCR positive samples, plus sewage sampling. During the peak of the pandemic, when COVID-19 positivity was high and Delta derivatives and Omicron variants were in circulation, GeSCOPE received over 30-50 RT-PCR positive samples daily from testing centers across Mumbai’s 24 wards. The turnaround time for sequencing was quick, and results were available locally within 72 h.
- *Workforce Readiness:* MDRL and VRDL at KH were already functional well before GeSCOPE was established and funded by MCGM and Indian Council of Medical Research. The laboratory trained scientists and technicians who were proficient in molecular diagnostics. Illumina supported the initial training of GeSCOPE’s personnel on their sequencers. Hence, GeSCOPE was launched in a state of readiness to take on PHG sequencing of SARS-CoV-2 samples.

### Sustainability

Sustainability is a critical factor for any public health entity. To address this issue, GeSCOPE has been integrated with MDRL, MCGM’s public health molecular diagnostic laboratory, and ICMR’s Viral Research & Diagnostic Laboratory, VRDL, all housed at KH. This is important as VRDL has an annual budget that is approved and independently funded by ICMR. This model also optimizes human resource utilization between MDRL, VRDL, and GeSCOPE. Current staffing includes two research scientists, one research assistant, two lab technicians, and trained support staff, who are available for PHG sequencing activities. In addition, GeSCOPE was established with funding to cover 5 years of operations.

However, this approach has its limitations. Today, at the 5-year mark, GeSCOPE is facing some funding gaps to cover certain materials, supplies, and operational costs. Therefore, GeSCOPE’s leadership is actively developing a diversified, multipronged, funding model to include NGOs, private philanthropies, and CSR support, along with MCGM’s support, for a stable support environment.

### Integration into public health policy and response

Currently, PCR diagnosis of infectious pathogens is performed regularly at MDRL and VDRL, coupled with pathogen sequencing data from GeSCOPE. The insights gained on genetic variations of the active infective pathogens and the heterogeneity in the pathogenic clinical presentations and disease severity are used by MCGM health policy experts to define and tailor the public health response to a particular pathogenic outbreak. These data also contribute to a better understanding of disease pathogenesis, allowing for informed public health policies and responses. The recent identification of Dengue subtypes and the rapid detection of drug susceptibility in multidrug-resistant *Mycobacterium tuberculosis* strains, has had a tangible impact on Mumbai’s ability to manage pathogenic outbreaks.

## Discussion

PHG is a recognized interdisciplinary field, bringing together genomic sequencing capacity, with populational science, behavioral science, and policy development to benefit public health. Integrating these elements into local public health systems, for rapid and effective public health policy decisions and actions needed during an infectious outbreak, becomes a challenge when PHG surveillance systems are not locally available, adequately distributed, or networked at a national level.

Mumbai’s case is an example of this challenge. Africa’s Centers for Disease Control and Prevention (ACDC) has a model where central and regional PHG centers are connected with their member nations’ national institutes of health, which in turn coordinate with local public health systems. Despite having a hub-and-spoke model, COVID-19 also challenged ACDC in its responsiveness to COVID-19 in several cities of its member nations. To address some of the key challenges, the leadership of the Africa CDC undertook a review and identified the need for greater local PHG capacity to drive an adequate crisis response (6).

The United Kingdom (UK), on the other hand, in March 2020, funded and established a robust national PHG network. The national COVID-19 Genomics UK (COG-UK) Consortium was developed between sixteen academic institutes, the public health agencies of England, Scotland, Wales, and Northern Ireland, national labs, national institutes, and close to eighty of the National Health Service trusts. This was a national system where sequencing capacity was broadly distributed and integrated with the public health systems at the level of the communities they served. In 2022, the Rand Corporation published its review report on COG-UK’s performance during COVID-19, outlining the great impact COG-UK had in advancing the scientific understanding of COVID-19, informing public health policy, guiding key public health decisions, and enhancing the UK’s PHG capacity in dealing with future pandemics (7).

However, Africa CDCs and COG-UK PHG regional networks are not the norm. PHG capacity, even at a country level, is heterogeneous. Before the COVID-19 pandemic, only 60.0% of countries performed PHG surveillance, 26.7% of countries performed limited surveillance, 13.3% of countries had no surveillance capacity, and the remaining countries had no data on surveillance (8).

### Lessons learned

Establishing critical PHG capacity was challenging, especially during a pandemic. Several elements were critical for the success, and many components have to integrate and work in synergy to succeed in a timely fashion. Our experience showed that the following generalizable lessons are necessary for success.

- Large metropolitan areas, especially megacities such as Mumbai, are particularly susceptible to infectious outbreaks and their rapid spread. Our experience and the lessons from other PHG systems underscore the need to build PHG capacity locally, especially in major population clusters, and integrate these into a national PHG network.
- While the need to develop PHG capacity is often recognized by many, there should be one entity that leads and catalyzes the effort, bringing in critical players who can make a difference.
- It is essential to rapidly establish an alliance of critical stakeholders, fully aligned around the cause and goals, and able to provide the elements necessary to establish PHG capacity. COG-UK’s example is a case in point. Lack of alignment, improper selection of alliance members, and not leveraging their strengths often unwinds such efforts.
- Assessing the readiness of a public health system to benefit from PHG capabilities, while also understanding the environment, the context, the challenges, and the limitations that will have to be dealt with, is vital for successfully establishing PHG capacity.
- Involving the public, private, social, and governmental sectors from the start in an aligned partnership is critical in such efforts. Too often, the social sector’s involvement is ignored. Lack of alignment with the community is an overlooked cause of failure.
- Building a successful alliance requires the definition of clear roles and responsibilities, empowering each alliance member to make decisions and act in their areas of responsibility while coordinating efforts across all alliance members.
- Creating a hierarchical or dominantly consensus-based alliance is often suboptimal. Our experience suggests that a federated structure, with autonomy and distributed decision-making, is often a more effective model.
- Properly defining and accessing adequate funding and doing so quickly is vital. Poorly developed funding plans, underfunding, funding delays, and poorly implemented funding structures often fail to get projects off the ground.
- Ensuring the sustainability of a PHG system over the long term, based on realistic business and sustainability plans, is essential. More often than not, the lack of properly developed and implemented business and sustainability plans is a cause of failure.
- Establishing a PHG system with clear and measurable outcomes, in realistic time frames, that are beneficial to all stakeholders, is another area that is not given adequate attention. Not doing so inevitably creates misalignment, fragmentation, and support challenges over the long term.
- It is essential that PHG capacity is effectively integrated into the existing public health system. The choice of where PHG is established in an existing public health system is a critical decision.

### Limitations

While our experience has many generalizable lessons as detailed above, extrapolating from our experience to other environments also has limitations. The success of our approach was predicated on creating strong alliances with well-established and complementary entities with a common purpose. We also benefited from significant resource and time commitments by all alliance members, which may not be possible in other situations. The MCGM and Mumbai’s public health leadership also extended their full support. This alignment between key public, private, and social stakeholders was critical. In many situations, the lack of alignment can become a serious impediment. Finally, we were fortunate that KH was already an established public health infectious disease hospital with trained personnel. MDRL and VRDL, supported by MCGM and Indian Council of Medical Research, were already actively functioning at KH and had been integrated into Mumbai’s public health system for some time. Institutional capacity, such as KH, would first need to be established for a PHG system to be successful. In the absence of this, the time and resources required to build such a system could become a major limitation.

## Conclusion

The establishment of GeSCOPE has transformed Mumbai’s ability to expeditiously identify, act upon, and contain infectious outbreaks, better protecting its citizens and more effectively managing outbreaks. As several cities and communities continue to face challenging infectious outbreaks, including new COVID-19 variants and the recent outbreaks of Dengue, Measles, Mpox, Ebola, Chikungunya, Oropouche (9), etc., we hope our experience and the lessons we learned can help guide others in establishing and strengthening their own PHG capacity.

## Data Availability

The original contributions presented in the study are included in the article/supplementary material, further inquiries can be directed to the corresponding authors.

## References

[1] Reid A. (2021). Where did the Delta variant come from and what will happen next? National Center Sci Educ. Available online at: https://ncse.ngo/where-did-delta-variant-come-and-what-will-happen-next (Accessed September 2, 2021).

[2] Novelli G Colona VL Pandolfi PP. A focus on the spread of the delta variant of SARS-CoV-2 in India. Indian J Med Res. (2021) 153:537–41. doi: 10.4103/ijmr.ijmr_1353_21, PMID: 34259195 PMC8555585

[3] Jha P Deshmukh Y Tumbe C Suraweera W Bhowmick A Sharma S . COVID mortality in India: national survey data and health facility deaths. Science. (2022) 375:667–71. doi: 10.1126/science.abm5154, PMID: 34990216 PMC9836201

[4] Kelly-Cirino CD Nkengasong J Kettler H Tongio I Gay-Andrieu F Escadafal C . Importance of diagnostics in epidemic and pandemic preparedness. BMJ Glob Health. (2019) 4:e001179. doi: 10.1136/bmjgh-2018-001179, PMID: 30815287 PMC6362765

[5] Eyre DW. Infection prevention and control insights from a decade of pathogen whole-genome sequencing. J Hosp Infect. (2022) 122:180–6. doi: 10.1016/j.jhin.2022.01.024, PMID: 35157991 PMC8837474

[6] Akello G. (2025). Pandemic preparedness in African countries: status quo and the way forward. Africa Policy & Research Institute. Available online at: https://afripoli.org/pandemic-preparedness-in-african-countries-status-quo-and-the-way-forward (Accessed March 24, 2025).

[7] Marjanovic S Romanelli RJ Ali GC Leach B Bonsu M Rodriguez-Rincon D . COVID-19 genomics UK (COG-UK) consortium: final report. Rand Health Q. (2022) 9:24. PMID: 36238008 10.7249/rhq-09-04-24 PMC9519096

[8] Yu H Chen Z Azman A Chen X Zou J Tian Y . Global landscape of SARS-CoV-2 genomic surveillance, public availability extent of genomic data, and epidemic shaped by variants. Res Sq. (2021) 29:rs.3.rs-927070. doi: 10.21203/rs.3.rs-927070/v1 [Preprint].

[9] Centers for Disease Control and Prevention Current Outbreak List, USA. (2025). Available at: https://www.cdc.gov/outbreaks/index.html

